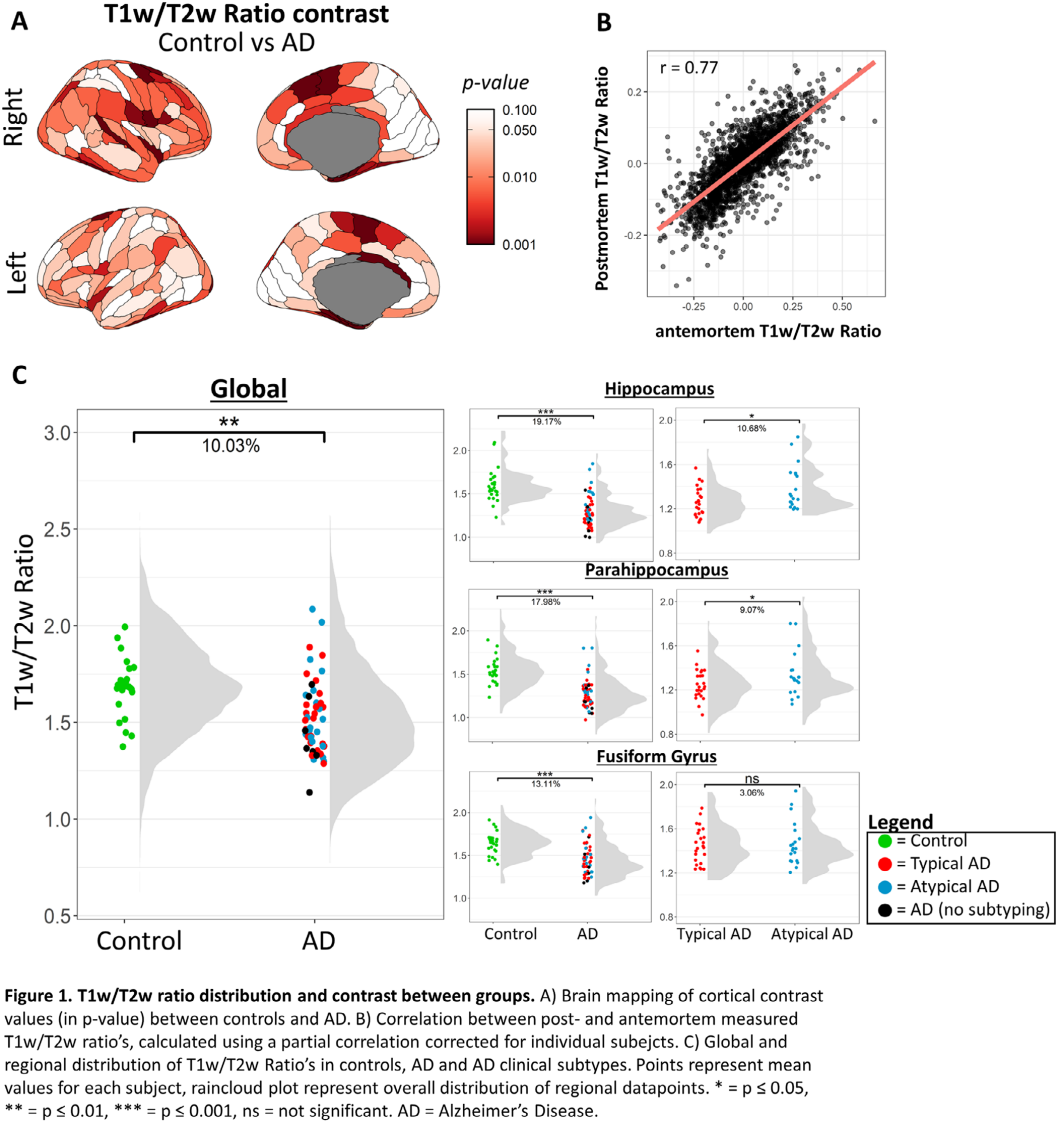# T1w/T2w Ratio reflects microstructural integrity in Alzheimer's Disease

**DOI:** 10.1002/alz70855_099349

**Published:** 2025-12-23

**Authors:** Niels Reijner, Danielle V Toen, Betty M. Tijms, Annemieke J.M. Rozemuller, Louise van der Weerd, Frederik Barkhof, Wilma D.J. Van de Berg, Laura E. Jonkman

**Affiliations:** ^1^ Amsterdam Neuroscience, Neurodegeneration, Amsterdam, Netherlands; ^2^ Amsterdam Neuroscience, Brain Imaging, Amsterdam, Netherlands; ^3^ Amsterdam UMC, location VUmc, Department of Anatomy and Neurosciences, Section Clinical Neuroanatomy and Biobanking, Amsterdam, Netherlands; ^4^ Leiden UMC, Leiden, Zuid‐Holland, Netherlands; ^5^ Alzheimer Center Amsterdam, Neurology, Vrije Universiteit Amsterdam, Amsterdam UMC location VUmc, Amsterdam, Noord‐Holland, Netherlands; ^6^ Department of Pathology, Amsterdam Neuroscience, Amsterdam UMC, Amsterdam, Noord‐Holland, Netherlands; ^7^ Institutes of Neurology & Healthcare Engineering, University College London, London, United Kingdom; ^8^ Amsterdam UMC, location VUmc, Amsterdam, Noord‐Holland, Netherlands; ^9^ Amsterdam UMC ‐ location VUmc, Amsterdam, Netherlands; ^10^ Amsterdam UMC, location VUmc, Amsterdam, Netherlands

## Abstract

**Background:**

Microstructural changes precede Alzheimer's‐related brain atrophy and cognitive decline by years, and persist throughout disease progression, making their detection crucial. The T1‐weighted/T2‐weighted MR image ratio (T1w/T2w ratio) can be a promising and clinically feasible tool for monitoring these changes, but its sensitivity to microstructural alterations is not fully understood. Here we aim to investigate its relationship with histologically measured microsctructural changes to clarify its potential as a neuroimaging biomarker for AD and its subtypes.

**Methods:**

Postmortem *in‐situ* 3T T1w and T2w MRI scans were obtained from 52 AD and 27 control brain donors. Antemortem 3T T1w and T2w MRI scans within 2 years from postmortem scans where collected when available (*n* = 9). Images were coregistered, bias‐field corrected, segmented, and processed into standardized T1w/T2w ratios and parcellated using the Brainnetome atlas, all in SPM12. Seven right‐hemisphere regions were immunostained for PLP (myelin), NfL (neuro‐axonal damage), IBA1 (microglia), Meguro (iron), Aβ, and pTau, with quantification via Qupath. immunoreactivity (area%) was calculated for all markers. Statistical models were adjusted for age, sex, postmortem delay, and intracranial volume when appropriate.

**Results:**

Compared to controls, postmortem AD brains showed a global decrease in T1w/T2w ratio (‐10.03%, *p=*0.001), regionally most pronounced in the hippocampus (‐19.17%, *p* <0.001), and parahippocampus (‐17.98%, *p* <0.001). (Para)hippocampal regions showed lower T1w/T2w ratio in typical AD compared to atypical AD (‐9.96%, *p* = 0.001). Postmortem and antemortem T1w/T2w ratios showed to be strongly correlated (*r* = 0.77, *p* <0.001). Myelin and microglia density were associated with T1w/T2w ratio in controls (myelin: β=0.17, *p* = 0.038; microglia: β=‐0.21, *p* = 0.003) and AD groups (myelin: β=0.31, *p* <0.001; microglia: β=‐0.39, *p* <0.001). In addition, only the AD group showed associations between T1w/T2w ratio and Aβ (β=0.31, *p* <0.001), pTau (β=‐0.16, *p* = 0.021), neuro‐axonal damage (β=0.20, *p* <0.001), and iron density (β=0.15, *p* = 0.036).

**Conclusions:**

These findings show that the T1w/T2w ratio is not solely a marker of myelin integrity, but a broader indicator of cortical tissue integrity in AD. Furthermore, the T1w/T2w ratio distinguishes control from AD brains and can regionally differentiate between clinical subtypes. This expanded understanding could improve its application in both research and clinical settings.